# Elucidating Interactions Between SARS-CoV-2 Trimeric Spike Protein and ACE2 Using Homology Modeling and Molecular Dynamics Simulations

**DOI:** 10.3389/fchem.2020.622632

**Published:** 2021-01-05

**Authors:** Sugunadevi Sakkiah, Wenjing Guo, Bohu Pan, Zuowei Ji, Gokhan Yavas, Marli Azevedo, Jessica Hawes, Tucker A. Patterson, Huixiao Hong

**Affiliations:** National Center for Toxicological Research, U.S. Food and Drug Administration, Jefferson, AR, United States

**Keywords:** SARS-CoV-2, spike protein, molecular dynamics simulations, homology modeling, COVID-19

## Abstract

Severe Acute Respiratory Syndrome Coronavirus-2 (SARS-CoV-2) causes coronavirus disease 2019 (COVID-19). As of October 21, 2020, more than 41.4 million confirmed cases and 1.1 million deaths have been reported. Thus, it is immensely important to develop drugs and vaccines to combat COVID-19. The spike protein present on the outer surface of the virion plays a major role in viral infection by binding to receptor proteins present on the outer membrane of host cells, triggering membrane fusion and internalization, which enables release of viral ssRNA into the host cell. Understanding the interactions between the SARS-CoV-2 trimeric spike protein and its host cell receptor protein, angiotensin converting enzyme 2 (ACE2), is important for developing drugs and vaccines to prevent and treat COVID-19. Several crystal structures of partial and mutant SARS-CoV-2 spike proteins have been reported; however, an atomistic structure of the wild-type SARS-CoV-2 trimeric spike protein complexed with ACE2 is not yet available. Therefore, in our study, homology modeling was used to build the trimeric form of the spike protein complexed with human ACE2, followed by all-atom molecular dynamics simulations to elucidate interactions at the interface between the spike protein and ACE2. Molecular Mechanics Poisson-Boltzmann Surface Area (MMPBSA) and *in silico* alanine scanning were employed to characterize the interacting residues at the interface. Twenty interacting residues in the spike protein were identified that are likely to be responsible for tightly binding to ACE2, of which five residues (Val445, Thr478, Gly485, Phe490, and Ser494) were not reported in the crystal structure of the truncated spike protein receptor binding domain (RBD) complexed with ACE2. These data indicate that the interactions between ACE2 and the tertiary structure of the full-length spike protein trimer are different from those between ACE2 and the truncated monomer of the spike protein RBD. These findings could facilitate the development of drugs and vaccines to prevent SARS-CoV-2 infection and combat COVID-19.

## Introduction

The Severe Acute Respiratory Syndrome Coronavirus 2 (SARS-CoV-2) was first identified in Hubei, China, and causes the severe respiratory syndrome known as COVID-19 in humans. Seven strains of human coronaviruses have been identified, which include human coronavirus HKU1 (HCoV-HKU1), human coronavirus 229E (HCoV-229E), human coronavirus NL63 (HCoV-NL63), human coronavirus OC43 (HCoV-OC43), severe acute respiratory syndrome coronavirus (SARS-CoV), Middle East respiratory syndrome-related coronavirus (MERS-CoV), and SARS-CoV-2 (Malik, 2020). Coronaviruses are composed of four genera belonging to the *coronaviridae family* (Zhang and Liu, 2020), wherein SARS-CoV-2, SARS-CoV, and MERS-CoV belong to the β-coronavirus genus (Petrosillo et al., 2020). Despite a high degree of structural homology with SARS-CoV and MERS-CoV (Jaimes et al., 2020), SARS-CoV-2 is much more transmissible than its predecessors, which may be attributable to unique differences in the spike protein (Rabaan et al., 2020). As of October 21, 2020, more than 1.1 million deaths and 41.4 million infected cases have been confirmed (https://www.worldometers.info/coronavirus/), with the COVID-19 pandemic remaining a significant global threat for eight continuous months and counting.

**Figure 1:**
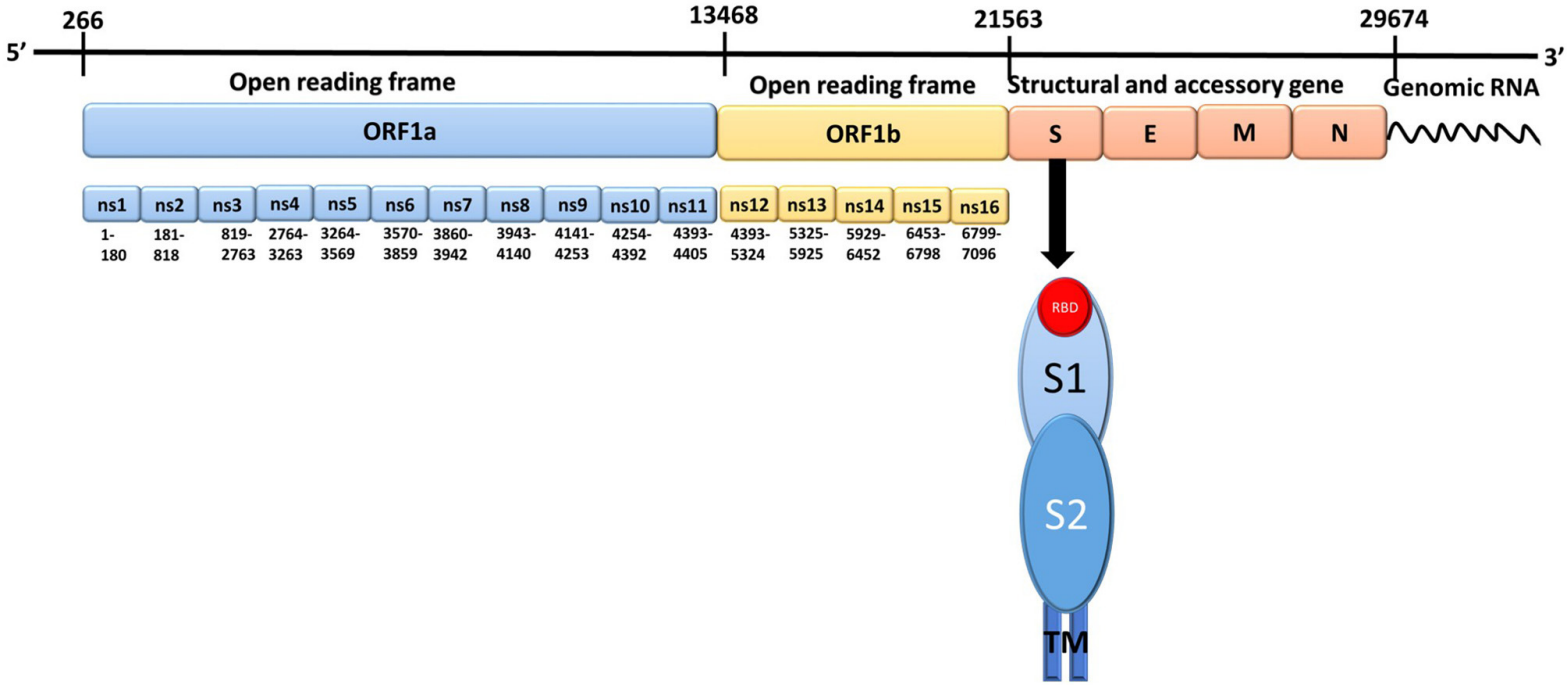
The genomic structure of Severe Acute Respiratory Syndrome Coronavirus-2. There are 14 open reading frames (ORFs) within the two primary transcriptional units ORF1a and ORF1b. S, Spike protein; E, Envelope protein; M, Membrane protein; N, Nucleocapsid protein; ns, Non-structural protein; RBD, Receptor binding domain; TM, Transmembrane region; S1, spike protein subunit 1; S2, spike protein subunit 2.

SARS-CoV-2 is a positive-sense, single-stranded RNA virus that encodes 16 non-structural proteins (NSP1-NSP16), four structural proteins (spike, membrane, envelope, and nucleocapsid), and nine accessory proteins (Figure 1; Romano et al., 2020). Spike proteins present on the virion surface are responsible for targeting host cells and triggering fusion of viral and host cell membranes, which are critical steps in initiating infection and enabling the transfer of viral RNA into host cells. Membrane and envelope proteins are responsible for the virus shape and assembly/budding, respectively. The nucleocapsid protein enters the host cell along with the SARS-CoV-2 genetic material, which serves to facilitate RNA transcription, replication, virus assembly, and release (Kang et al., 2020; Zeng et al., 2020). Since the spike protein plays a major role in initializing viral infection through binding to ACE2, inhibiting the binding of the spike protein to ACE2 is an attractive strategy for developing drugs to block the spread of SARS-CoV-2 infection and treat COVID-19 (Das et al., 2020; Wu et al., 2020). Therefore, understanding interactions between the spike protein and ACE2 may facilitate the development of drugs that target binding of the spike protein to ACE2.

The spike protein contains 1273 amino acids and is composed of two subunits, S1 (amino acids 14-685) and S2 (amino acids 686-1273), which are responsible for receptor binding and membrane fusion with the host cell, respectively, preceded by a short signal peptide (amino acids 1-13). The S1 subunit consists of three domains: an N-terminal domain (NTD; amino acids 14-305), a receptor binding domain (RBD; amino acids 319-541), and a carboxy-terminal domain (CTD) which has two subdomains (SD1 and SD2) (Henderson et al., 2020; Huang et al., 2020; Tang et al., 2020a,b). The S2 subunit consists of a fusion peptide (FP; amino acids 788-806) composed of hydrophobic residues, heptapeptide repeat 1 (HR1; amino acids 912-984), heptapeptide repeat 2 (HR2; amino acids 1163-1213), a transmembrane domain (TM; amino acids 1213-1237), and a cytoplasmic domain (CP; amino acids 1237-1273) (Astuti and Ysrafil, 2020; Huang et al., 2020; Tang et al., 2020a,b). In its native form, the spike protein is present as a trimer on the surface of the virion, with the S1 and S2 subunits forming the extracellular stalk and bulbous “crown,” for which the Latin translation is “corona” (Huang et al., 2020; Tang et al., 2020a,b; Walls et al., 2020). The crown of the trimeric spike protein undergoes hinge-like conformational changes between a closed/down conformation and a less-stable open/up conformation (Huang et al., 2020; Tang et al., 2020a,b; Walls et al., 2020; Wrapp et al., 2020). In the open/up conformation, the RBD is accessible for binding to the ACE2 receptor; whereas, in the closed/down conformation, the RBD cannot interact with the ACE2 receptor (Ortega et al., 2020; Wrapp et al., 2020). Upon binding to the ACE2 receptor, the spike protein trimer undergoes a conformational change resulting in the accessibility of the S1 and S2 cleavage sites to host proteases (Lan et al., 2020; Shang et al., 2020; Xia et al., 2020). Cleavage of the S1 and S2 subunits primes the spike protein for membrane fusion by enabling insertion of the S2 FP domain into the host cell membrane, which enables subsequent interactions between the HR1 and HR2 coiled-coil domains to form a six helical bundle (6-HB). This bundle stabilizes another S2 subunit conformational change in which viral and host membranes are close enough in proximity to trigger membrane fusion (Huang et al., 2020; Tang et al., 2020a,b).

Recently, structures of the trimeric form of mutant or truncated spike proteins complexed with antibodies were reported. Walls et al. determined atomic models of SARS-CoV-2 spike protein in the closed/down (6VXX) trimeric conformation and a one-up (6VYB) trimeric conformation, in which a single S unit is in the open/up conformation and two S units are in the closed/down conformation (Walls et al., 2020). However, the full-length wild-type S protein was not determined due to missing residues and loops, and spike protein trimers bound to the ACE2 receptor were not evaluated. Thus, a crystal structure of the wild-type trimeric form of spike protein bound to ACE2 has not yet been determined (Song et al., 2018).

In this study, a complex structure of the wild-type trimeric spike protein with ACE2 was constructed using homology modeling based on the atomic details of the trimeric form of the spike protein with the one-up conformation and the RBD of the spike protein with ACE2. The homology models were evaluated using a Ramachandran plot and the highest quality model was subjected to molecular dynamics simulations to identify interactions between the trimeric spike protein and ACE2.

## Methods and Materials

### Homology Modeling

The primary sequence of the spike protein (ID: P0DTC2) was retrieved from the UniProt database (https://www.uniprot.org) and used as a template sequence. The mutant trimeric spike protein of SARS-CoV-2 with the one-up conformation (PDB ID: 6VYB) (Walls et al., 2020) and the complex of spike protein RBD of SARS-CoV-2 with ACE2 (PDB ID: 6M0J) (Lan et al., 2020) were selected as the template structures. Sequence alignment between templates and targets was performed using the EBI-Clustal Omega Server (Madeira et al., 2019). Homology modeling was performed using the Modeler v9.24 (Sali and Blundell, 1993) program. Ten models were generated, and one model was selected based on the DOPE energy value. To avoid steric clashes, the selected model was energy minimized using the Schrödinger suite (www.schrodinger.com) and evaluated for its stereo-chemical quality using a Ramachandran plot (https://swissmodel.expasy.org/assess).

### Molecular Dynamics Simulations

The validated homology model of the trimeric form of the spike protein complexed with ACE2 was used as the starting structure for molecular dynamics simulations using Amber 18 (https://ambermd.org/CiteAmber.php). The *tleap* from AmberTools was used to prepare topologies and coordination files for the protein using protein.ff18SB forcefield (Ponder and Case, 2003; Maier et al., 2015) by adding the force field and hydrogen atoms. The prepared system was then placed inside an octahedral box 10 Å away from the protein surface, solvated with the TIP3P (Jorgensen et al., 1983) water model, and subjected to energy minimization. The counter ions were added to neutralize the unbalanced charge of the system. The whole process was divided into 2 phases. In phase 1, the solute molecules were constrained and only the solvents were minimized and equilibrated. Subsequently, the steepest descent method (1,000 steps) followed by conjugate gradient (4,000 steps) methods were applied to minimize the whole system (solute and solvent). The minimized system was gradually heated from 0 to 310 K, and the entire system was equilibrated without any constraints. During the simulations, the system temperature and pressure were maintained at 310.5 K and 1 atm, respectively. In Phase 2, a 100 nanosecond (ns) unrestrained production run was applied to the system. The SHAKE (Ryckaert et al., 1977) algorithm and Particle Mesh Ewald (PME) (Darden et al., 1993) were applied for the hydrogen bonds and the long-range electrostatic interactions, respectively. Coordinate files were saved for every 5 picosecond (ps). The resultant trajectory was processed using the CPPTRAJ (Roe and Cheatham, 2013) from AmberTool package, Visual Molecular Dynamics (Humphrey et al., 1996), and PyMol (https://pymol.org). The kclust algorithm from MMTSB toolset (http://www.mmtsb.org/) was used to cluster the resultant trajectory. The representative structure from the cluster analysis was used for hydrogen bond and energy analysis. The hydrogen bond between the trimeric form of the spike protein and ACE2 was determined using a distance cut-off value of 4 Å or less.

### Binding Free Energy Calculation

The spike protein, ACE2, and spike protein-ACE2 complex structures were used to calculate binding free energy. The *tleap* from AmberTools18 (https://ambermd.org/AmberTools.php) was used to generate the gas phase, solvated complex topology (prmtop); and coordination (inpcrd) files for the spike protein, ACE2, and spike protein-ACE2 complex. The MMPBSA.py (Miller et al., 2012) script from the Amber package was used to calculate the binding free energy using the Molecular Mechanism-Generalized Born Surface Area (MMGBSA) approach. The representative structure from clustering analysis was used to calculate binding free energies using Equation (1)

(1) $$\begin{array}{l} \Delta G_{BFE}=G_{S-ACE2}-G_{S}-G_{ACE2} \end{array}$$

where Δ*G*_*BFE*_ represents the binding free energy between the spike protein and ACE2. The terms *G*_*S*−*ACE*2_, *G*_*S*_, and *G*_*ACE*2_ represent the free energy of the spike protein-ACE2 complex, spike protein, and ACE2, respectively. The MMPBSA.py script was used to calculate interaction and solvation free energies for the spike protein, ACE2, and spike protein-ACE2 complex. The energy values were calculated using Equation (2)

(2) $$\begin{array}{l} \Delta G=\Delta G_{gas}+\Delta G_{sol}-T\Delta S \end{array}$$

where Δ*G*_*gas*_, Δ*G*_*sol*_, and *T*Δ*S* represent the gas phase molecular mechanism component, solvation of binding free energies, and changes in entropy due to ACE2 binding, respectively. Because our goal was to obtain an estimated free energy rather than an absolute value, and since the computational cost was high, entropy changes in the free energy calculation were ignored.

The gas phase molecular mechanism (Δ*G*_*gas*_) and solvation free energy (Δ*G*_*sol*_) were calculated using Equations (3–5)

(3) $$\begin{array}{l} \Delta G_{gas}=\Delta G_{ele}+\Delta G_{vdW} \end{array}$$

(4) $$\begin{array}{l} \Delta G_{sol}=\Delta G_{pol.sol}+\Delta G_{nonpol.sol} \end{array}$$

(5) $$\begin{array}{l} \Delta G_{non-polar}=\gamma *SASA+\beta \end{array}$$

where Δ*G*_*vdW*_ and Δ*G*_*ele*_ correspond to van der Waals and electrostatic interactions, respectively, and Δ*G*_*pol*.*sol*_ and Δ*G*_*nonpol*.*sol*_ represent polar solvation and non-solvation terms from the MMGBSA approach. The terms SASA, γ, and β denote solvent-accessible surface area, surface tension, and regression offset of the linear relationship, respectively. Default values were used to calculate the estimated binding free energy between the spike protein and ACE2.

### Interaction Free Energy of Residues

The representative structure from clustering analysis was used to analyze interaction free energy for residues at the interface of the spike protein and ACE2 using the *decomp* (Miller et al., 2012) module from the MMPBSA.py program. Contributions of the interacting residues were calculated based on the following energy terms: electrostatic contribution, non-polar solvation contribution, van der Waals contribution, and polar solvation, using Equation (6).

(6) $$\begin{array}{l} \Delta G_{residue}=\Delta G_{ele}+\Delta G_{nonpol.sol}+\Delta G_{vdW}+\Delta G_{polar} \end{array}$$

### Alanine Scanning

The selected interacting residues were mutated individually in the representative structure from the cluster analysis. The free energies of the mutated trimeric spike protein and ACE2 complexes were calculated by MMPBSA.py using Equation (7).

(7) $$\begin{array}{l} \Delta G_{BFE}=G_{S_{mut}-ACE2}-G_{S_{mut}}-G_{ACE2} \end{array}$$

where Δ*G*_*BFE*_ represents the estimated binding free energy for mutated spike protein and ACE2. The *G*_*S*_*mut*_−*ACE*2_, *G*_*S*_*mut*__, and *G*_*ACE*2_ terms represent the free energy components estimated for the mutated spike protein-ACE2 complex, mutated spike protein, and ACE2, respectively. The MMPBSA.py script was used to calculate the free energies for the mutated spike protein, ACE2, and mutated spike protein-ACE2 complex.

## Results and Discussion

### Homology Modeling of the Trimeric Form of the Spike Protein

**Figure 2:**
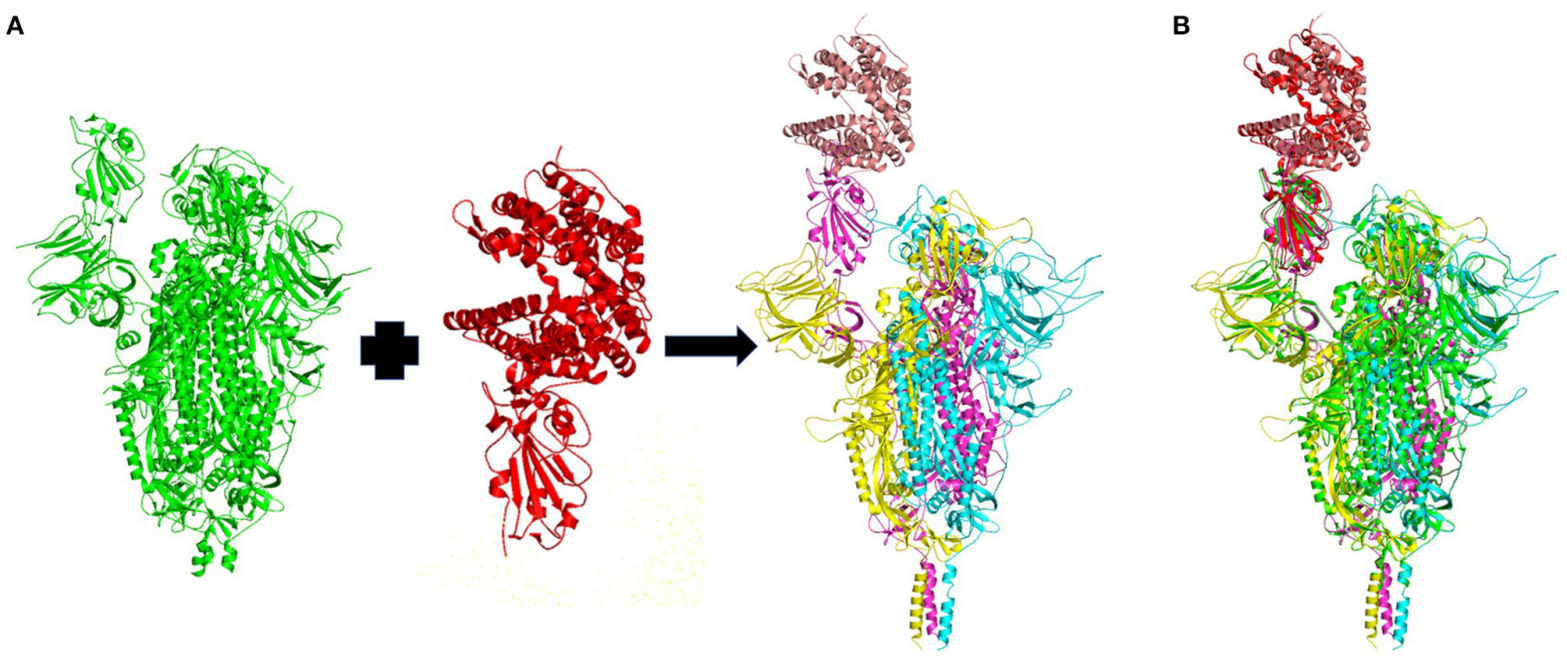
**(A)** Homology model of the wild-type trimeric form of Severe Acute Respiratory Syndrome Coronavirus-2 spike protein complexed with ACE2. The template structures are in green and red. The chain A, chain B, and chain C of the trimeric spike protein, and ACE2 are colored in yellow, magenta, cyan and brown, respectively. **(B)** Superimposition of the template structures (green and blue) and the modeled trimeric form of the spike protein (yellow, magenta, and cyan) complexed with ACE2 (brown).

Ten homology models of the trimeric form of the spike protein bound to ACE2 were generated and their DOPE energy values were calculated (Table 1). Model_4 showed the lowest DOPE energy value and was selected as the best structure for the trimeric form of spike protein complexed with ACE2. Model_4 was evaluated for stereochemical chemical quality using a Ramachandran plot. According to the Ramachandran plot (Supplementary Figure 1), 93.57 and 1.53% of residues were in the favored and outlier regions, respectively. Chain A, chain B, and chain C showed 94, 93.39, and 92.95% residues in the favored regions, respectively. Superimpositions between Model_4 and the reported structure of the trimeric form of the spike protein with the one-up conformation, and between Model_4 and the crystal structure of the truncated spike protein RBD bound with ACE2 (Figure 2), showed RMSD values of 0.28Å and 0.744 Å, respectively. This finding indicates that the homology model was consistent with the empirical structures. Therefore, Model_4 was used for subsequent molecular dynamics simulations.

**Table 1:** The DOPE energy values for homology models of the trimeric form of the Severe Acute Respiratory Syndrome Coronavirus-2 spike protein bound to ACE2.

**Model number**	**DOPE score**
Model_1	−453061.63
Model_2	−452078.13
Model_3	−452595.94
Model_4	−453871.81^*^
Model_5	−453290.16
Model_6	−452068.91
Model_7	−453414.09
Model_8	−452842.60
Model_9	−452185.50
Model_10	−452439.75

* *The lowest energy*.

### Molecular Dynamics Simulations

**Figure 3:**
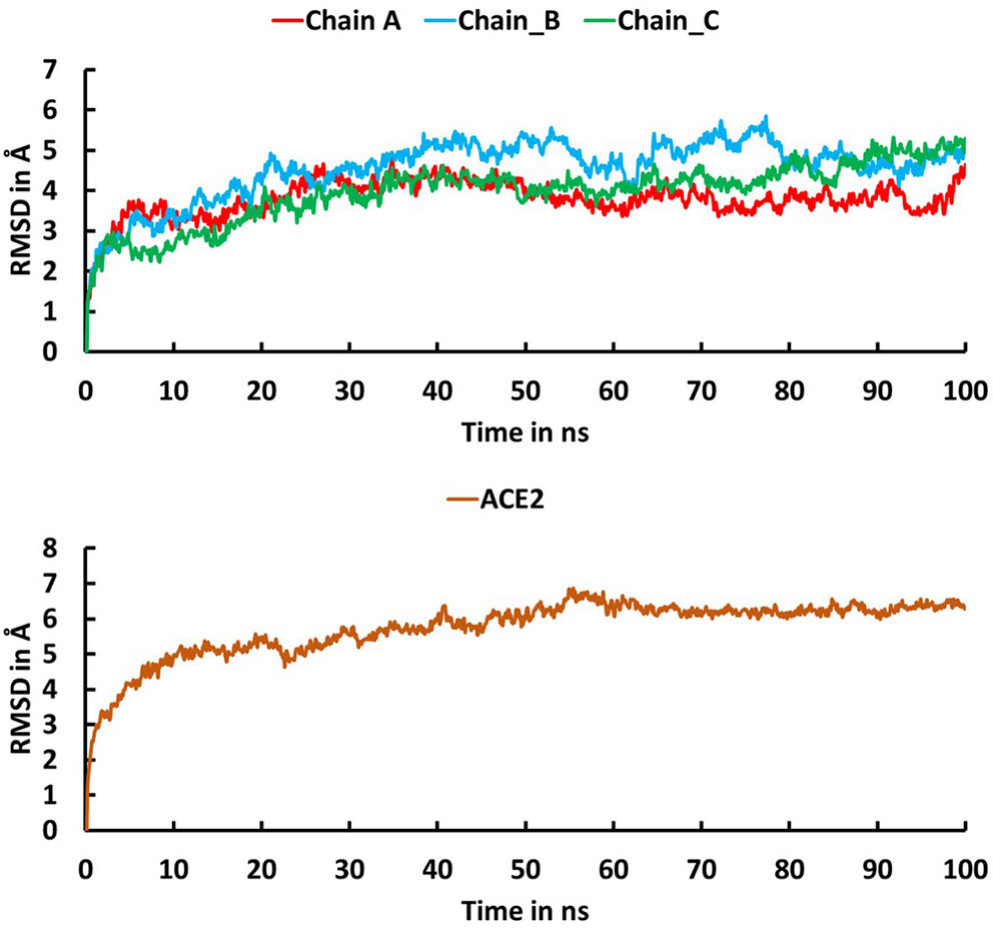
Root mean square deviation (RMSD) plot for the trimeric form of Severe Acute Respiratory Syndrome Coronavirus-2 spike protein (Chain A in red, Chain B in cyan, Chain C in green) and ACE2 (brown) during the 100 ns molecular dynamics simulations. The X-axis indicates time in ns and the Y-axis represents RMSD values in Å.

**Figure 4:**
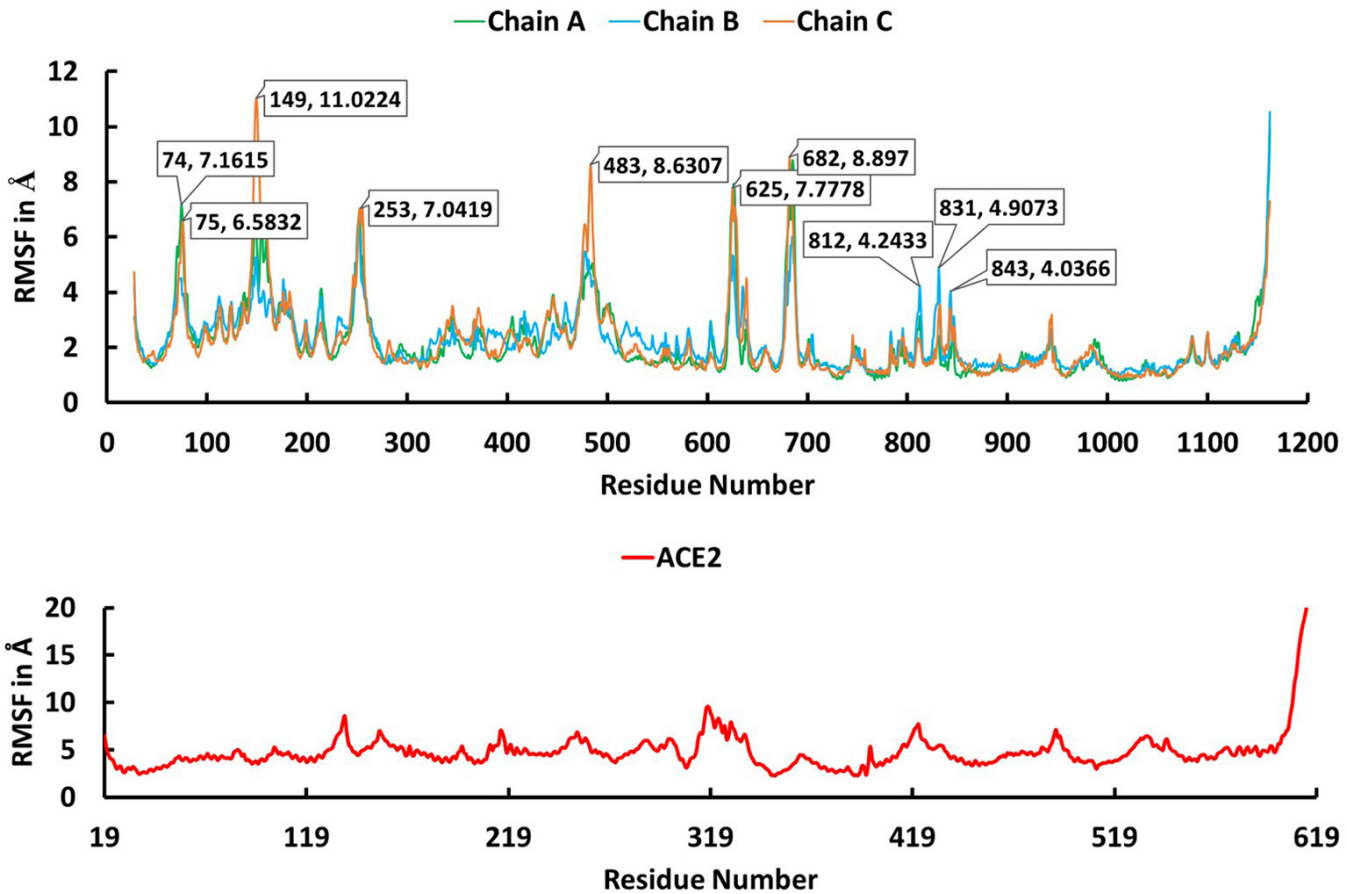
The root mean square fluctuation (RMSF) plot for Cα atoms in chain A (green), B (cyan), and C (brown) of the trimeric form of Severe Acute Respiratory Syndrome Coronavirus-2 spike protein and ACE2 (red) during the 100 ns molecular dynamics simulations. The X axis indicates residue number and Y axis represents RMSF value in Å.

The trajectory obtained from the molecular dynamics simulations was analyzed to identify critical residues at the interface between spike protein and ACE2. Stabilities of the trimeric form of the spike protein complexed with ACE2 and fluctuation of residues in the simulations were examined using root mean squared deviation (RMSD) of Cα atoms and root mean square fluctuation (RMSF), respectively. The RMSD plot (Figure 3) indicates that the trimeric form of wild-type spike protein was stable in the last 20 ns with a converged RMSD of ~5 Å. The averaged RMSD values for the chain A, chain B, and chain C were 3.36 Å, 4.51 Å, and 4.27 Å, respectively. ACE2 was stable in the last 40 ns with an average RMSD value of 5.74 Å. The high RMSD values are caused by the loop regions in the spike protein and ACE2. The RMSF plot (Figure 4) suggests that the residues in the secondary structure regions were quite stable, but the residues in the loop regions showed large fluctuations with RMSF values >3Å.

### Clustering Analysis

**Figure 5:**
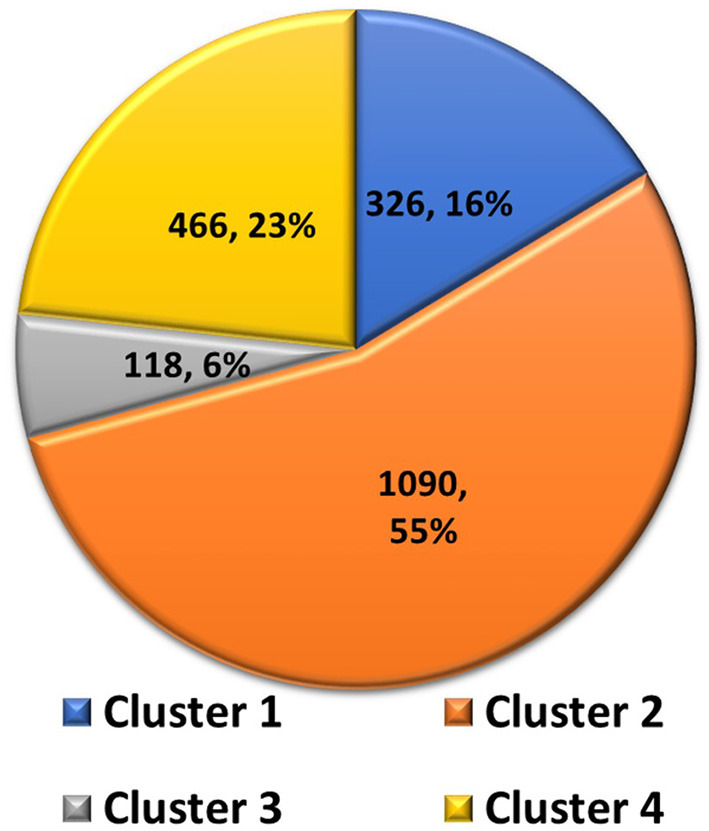
Pie chart of statistics of the 4 clusters of Severe Acute Respiratory Syndrome Coronavirus-2 spike protein complexed with ACE2. Number of structures and percentages are indicated in each pie segment.

Clustering analysis was used to select a representative structure from the trajectory for binding free energy analysis. The kclust algorithm from MMTSB was used to cluster the structures in the trajectory. The kclust is a fast and sensitive algorithm and is widely used to cluster large size proteins. First, structures were extracted from the trajectory file with an interval of five frames and saved as PDB files for clustering analysis. The extracted structures were then put into the kclust algorithm with the radius set to 3 Å. Four clusters were obtained from kclust that resulted in 326, 1,090, 118, and 466 structures, as shown in Figure 5. As Cluster two was the largest, the structure with the smallest RMSD value in Cluster two exhibited the shortest distances to other structures and, thus, was selected as the representative structure for subsequent binding free energy analysis via alanine scanning.

### Interacting Residues

**Figure 6:**
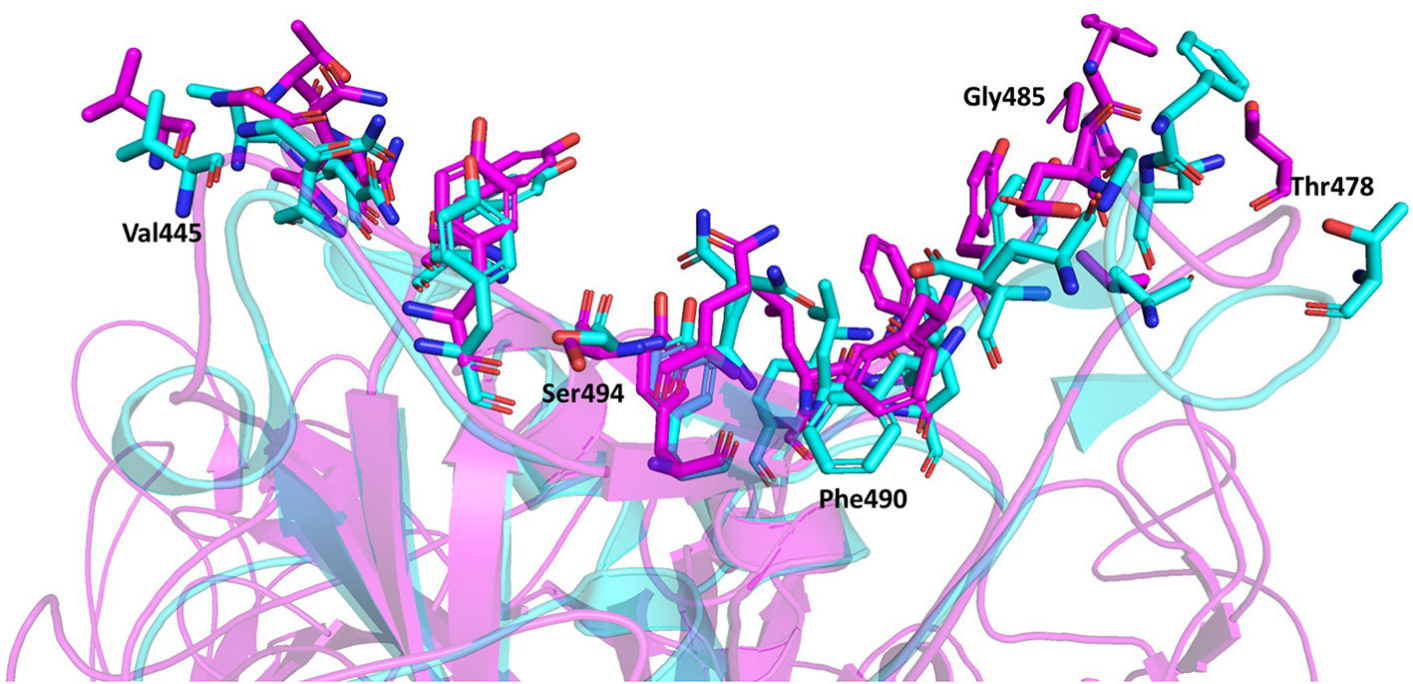
Superimposition of the spike protein-ACE2 complexes using the full-length trimeric spike protein (Magenta) and the truncated spike protein RBD monomer (Cyan). The interacting residues are represented by stick model illustrations, while the rest of the ACE2 proteins are depicted in ribbon model form. The five new interacting residues identified using the full-length trimeric spike protein complexed with ACE2 are labeled.

The representative structure from the cluster analysis was used to identify the residues between the trimeric spike protein and ACE2 at distances of 4 Å or less (Lan et al., 2020). Twenty residues of the trimeric spike protein were within 4 Å to ACE2 and were defined as interacting residues. Whereas, Lan et al. reported 17 spike protein RBD interacting residues within 4 Å to ACE2 when the truncated RBD monomer was analyzed. The superimposition of both complexes, the truncated spike protein RBD monomer complexed with ACE2 and the full-length trimeric spike protein complexed with ACE2, is depicted in Figure 6. Both approaches to identify interacting residues, using the full-length trimer described here and the truncated RBD monomer (Lan et al., 2020), resulted in identification of 14 conserved residues with distances to ACE2 of ≤4 Å. Lan et al. reported three additional interacting residues (Lys417, Tyr453, and Ala475) that were within 4 Å to ACE2 when using the truncated spike protein RBD monomer, but were slightly >4 Å to ACE2 in assessments using the full-length trimeric spike protein. However, given that their distances to ACE2 are very close to 4 Å using the full-length trimeric spike protein, it is acceptable to consider all three as interacting residues based on both approaches. Conversely, Glu484 was slight >4 Å from ACE2 using the truncated spike protein RBD monomer with a distance 4.39 Å but was <4 Å to ACE2 using the full-length trimeric spike protein. Thus, it is also acceptable to include Glu484 as an interacting residue according to both approaches. Therefore, a total of 18 ACE2-interacting residues may be considered to be conserved between both approaches using either the full-length trimer or the truncated RBD monomer. However, five additional interacting residues (Val445, Thr478, Gly485, Phe490, and Ser494) with distances <4 Å from ACE2 were identified using the full-length trimeric spike protein but were too far away to interact with ACE2 (5.69 Å, 7.62 Å, 5.72 Å, 5.34 Å, and 6.25 Å, respectively) using the truncated spike protein RBD monomer. Among these five new residues, four are in loop regions and the remaining one, Ser494, is at the end of a beta sheet in the spike protein RBD. Altogether, these data suggest that there are likely to be 23 spike protein residues capable of interacting with ACE2. These findings further suggest that the specific interacting residues may change based on the tertiary conformation, as indicated by the conformation difference between the full-length trimeric spike protein and its truncated RBD.

### Hydrogen Bond Interactions

**Figure 7:**
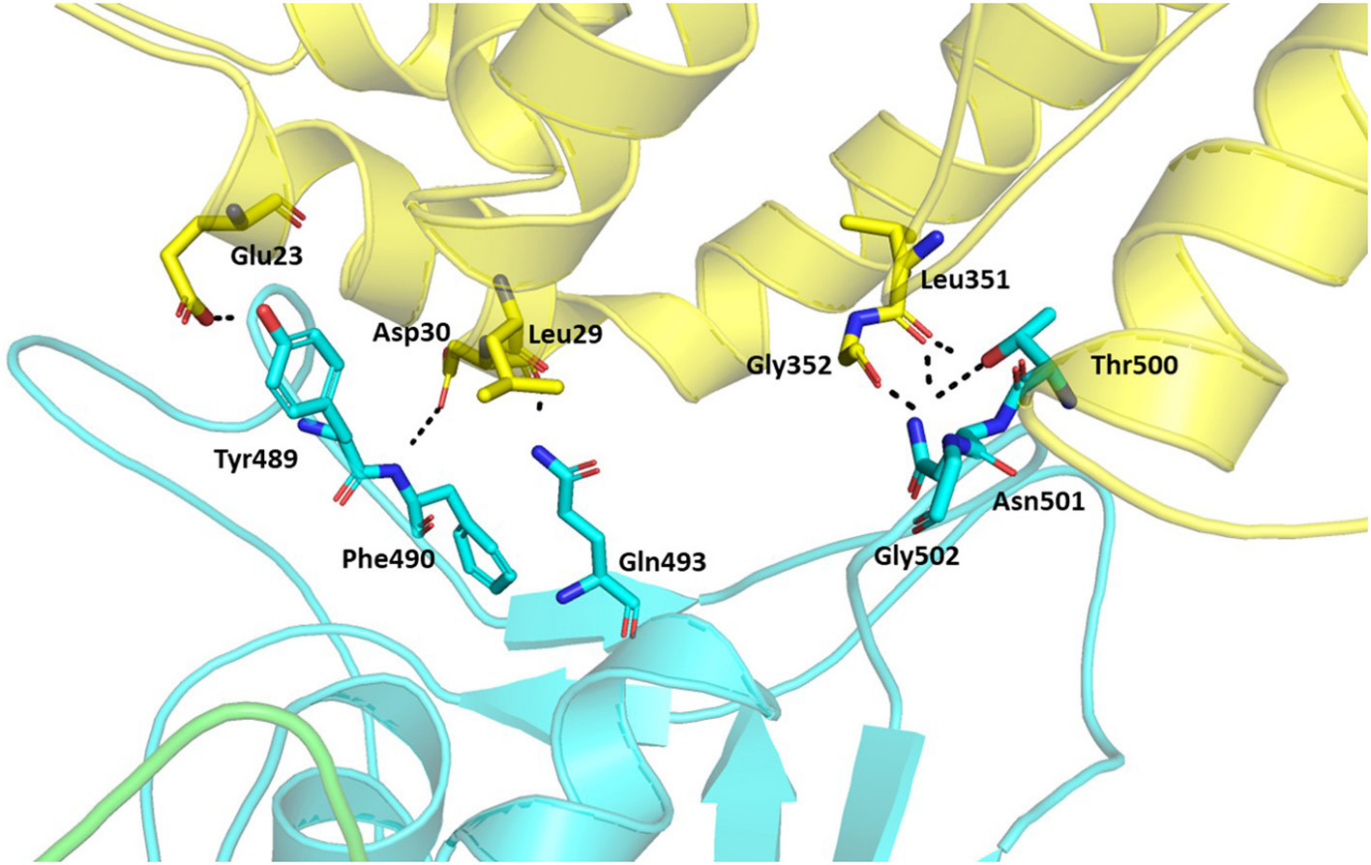
Hydrogen bonds between the trimeric spike protein and ACE2. The residues involved in the hydrogen bond formation are shown in stick. The trimeric spike (Chain A–Green and Chain B–Cyan) and ACE2 (Yellow) are shown in ribbons.

Hydrogen bonds are major interactions between proteins. Hence, hydrogen bonds formed between the spike protein and ACE2 in the representative structure from the molecular dynamics simulations were identified using CPPTRAJ from AmberTools. A hydrogen bond was formed if the distance between two hydrophilic atoms (O or N) near a hydrogen atom was <4 Å and the angle from the hydrogen to the two hydrophilic atoms was >135°. Using this criterion, hydrogen bonds were formed for six residues of the spike protein and five residues of ACE2 at the interface region (Table 2, Figure 7). Among these, five spike residues and one ACE2 residue were reported to form hydrogen bonds in the crystal structure of the truncated spike protein RBD complexed with ACE2 (Lan et al., 2020). Two residues that were reported to form hydrogen bonds in the crystal structure of the spike protein RBD bound with ACE2 (Lan et al., 2020), Leu455 and Phe456, did not form hydrogen bonds in our structure of the trimeric form of spike protein complexed with ACE2. On the other hand, Phe490 involved hydrogen bonding in our structure, but was not reported to form hydrogen bonds in the crystal structure of the truncated RBD of spike protein monomer complexed with ACE2. Thus, these findings suggest that the comprehensive hydrogen bonding network between the spike protein and ACE2 is likely to depend on the trimer of full-length spike protein complexed with ACE2.

**Table 2:** The residues involved in the hydrogen bond interactions between the trimeric form of the Severe Acute Respiratory Syndrome Coronavirus-2 spike protein and ACE2 complex.

**Residue in spike**	**Residue in ACE2**	**Distance Å**
Tyr489^*^	Glu23	2.715
Gln493^*^	Leu29	3.033
Phe490	Asp30^*^	3.184
Thr500^*^	Leu351	2.938
Asn501^*^	Leu351	2.909
Gly502^*^	Gly352	2.834

* *indicates the residues reported in the crystal structure of receptor binding domain of spike protein complexed with ACE2*.

### Binding Free Energy Calculation

MMPBSA.py script from AmberTools is a fast method to compute binding free energy compared to other methods (Marimuthu et al., 2020), such as Replica-Exchange Free-Energy Perturbation (Fratev and Sirimulla, 2019) and umbrella sampling (Kumar et al., 1992). MMPBSA.py script was used to calculate binding free energy values between the spike protein and ACE2 for the representative structure and its mutated structures from alanine screening. The estimated binding free energy between the spike protein and ACE2 was −60.54 kcal/mol, indicating that the spike protein tightly binds with ACE2. The contributions from different energy terms (van der Waals, electrostatic, polar and non-polar solvation, solvation, and gas phase) to the free energy of the spike protein-ACE2 complex, the spike protein, and ACE2 were calculated and are shown in Table 3. Electrostatic interactions contributed more to the total binding free energy than van der Waals. To investigate contributions of individual residues to the binding between the spike protein and ACE2, decomposition of free energies to individual residues was conducted. The energy components (van der Waals, electrostatic, and polar and non-polar solvation energy) of the 20 interacting residues with distances <4 Å from ACE2 are depicted in Table 4 and indicate that van der Waals interactions were the major force by which the interacting residues interacted with ACE2.

**Table 3:** MMPBSA energy values for the trimeric form of Severe Acute Respiratory Syndrome Coronavirus-2 spike protein bound with ACE2.

**Energy component**	**Spike-ACE2 complex**	**Spike**	**ACE2**	****Δ**G kCal/mol**
vdW	−31291.7513	−26670.0359	−4534.5528	−87.1626
EEL	280175.1068	−238713.6906	−40760.4637	−700.9525
EGB	−43799.2943	−33677.4334	−10860.3462	738.4853
ESURF	1395.7146	1141.7286	264.8911	−10.9051
G gas	−36997.8641	−35045.8034	−1163.9455	−788.1152
G solv	−42403.5797	−32535.7048	−10595.4551	727.5802
Total	−79401.4438	−67581.5082	−11759.4006	−60.5350

*vdW, van der Waals; EEL, Electrostatic energy; EGB, Polar solvation energy; ESURF, Non-polar solvation energy; G solv, Total solvation free energy; G gas:, Total gas phase free energy*.

**Table 4:** The 20 interacting residues from the decomposition analysis of Severe Acute Respiratory Syndrome Coronavirus-2 spike protein—ACE2 complex and their energy.

**Residue**	**van der Waals**	**Electrostatic**	**Polar solvation**	**Non-polar solvation**	**Total**
Val445	−0.311	−2.402	2.477	−0.113616	−0.349616
Gly446	−0.385	1.558	−1.172	−0.1263888	−0.1253888
Leu455	−0.971	−0.862	1.079	−0.1799856	−0.9339856
Thr478	−0.419	−0.473	0.702	−0.1375488	−0.3275488
Glu484	−0.822	100.71	−99.548	−0.2132928	0.1267072
Gly485	−1.044	0.466	0.272	−0.1362312	−0.4422312
Phe:486	−6.396	−3.798	4.073	−1.11034	−7.23134
Tyr:489	−4.958	−11.682	10.557	−0.55015	−6.63315
Gly496	−0.412	0.483	−0.12	−0.0960624	−0.1450624
Gln498	−1.528	2.017	−0.13	−0.29088	0.06812
Thr:500	−3.922	−4.18	4.737	−0.71958	−4.08458
Asn:501	−2.079	−9.038	7.484	−0.12496	−3.75796
Tyr:505	−3.503	−2.343	3.597	−0.57291	−2.82191
Gln:493	−2.628	−2.711	3.95	−0.52306	−1.91206
Gly:502	−0.508	−3.913	2.715	−0.19863	−1.90463
Phe:456	−1.989	−0.354	1.033	−0.3534	−1.6634
Tyr:449	−2.24	−2.808	4.02	−0.53238	−1.56038
Phe:490	−0.879	0.908	−1.492	−0.09493	−1.55793
Asn:487	−1.824	−4.005	4.825	−0.16806	−1.17206
Ser:494	−0.428	−0.841	0.254	−0.10336	−1.11836

The 20 interacting residues with distances <4 Å from ACE2 were used for alanine scanning. The current version of alanine scanning in MMPBSA script does not support energy calculation for the mutation of Gly to Ala. Hence, the four Gly residues were excluded from the alanine scanning calculation. Each of the remaining 16 interacting residues were mutated to alanine to generate a complex, then the free energy values were calculated for the complex using MMPBSA.py script. The binding free energy values of the wild-type and 16 mutated trimeric spike protein-ACE2 complexes were calculated and are reported in Table 5. Of the 16 alanine mutations, 15 resulted in structures with higher binding free energy than the representative wild-type structure, indicating that these 15 residues are likely to be important for spike protein binding to ACE2. The remaining one residue (Glu: 484) led to a structure with a slightly lower binding free energy, indicating that this residue is less important for the interaction between the spike protein and ACE2. Overall, the alanine screening analysis confirmed that the 20 interacting residues identified using the full-length trimeric spike protein play key roles in the binding interaction between the spike protein and ACE2.

**Table 5:** Binding free energy values for the representative and mutated Severe Acute Respiratory Syndrome Coronavirus-2 spike protein-ACE2 complexes.

**Residue mutated**	**Spike**	**ACE2**	**Spike-ACE2 complex**	**ΔG**
Tyr:489:Ala	−67555.8	−11759.4	−79366.7	−51.48
Phe:486:Ala	−67585.2	−11759.4	−79396.3	−51.64
Tyr:505:Ala	−67567.4	−11759.4	−79382.4	−55.61
Gln:493:Ala	−67521.5	−11759.4	−79337.1	−56.21
Asn:501:Ala	−67505.7	−11759.4	−79321.5	−56.41
Thr:500:Ala	−67555	−11759.4	−79372	−57.53
Tyr:449:Ala	−67557.1	−11759.4	−79374.2	−57.74
Ser:494:Ala	−67557.1	−11759.4	−79374.2	−57.74
Phe:456:Ala	−67572.5	−11759.4	−79387.6	−57.76
Lys:455:Ala	−67567.5	−11759.4	−79385.9	−58.95
Thr:478:Ala	−67557.5	−11759.4	−79376.6	−59.65
Asn:487:Ala	−67502.3	−11759.4	−79321.3	−59.65
Gln:498:Ala	−67520.7	−11759.4	−79340.1	−60.04
Phe:490:Ala	−67588.4	−11759.4	−79408.2	−60.41
Val:445:Ala	−67572.2	−11759.4	−79391.8	−60.25
Glu:484:Ala	−67522.4	−11759.4	−79342.9	−61.09

*ΔG: Binding free energy, ΔΔG: binding energy difference between the representative and mutated complex structure*.

Recently, 35,000 *de novo* hACE2 decoys were designed and CTC-445.2 was found to tightly bind with the RDB of the trimeric spike protein in four different states (state 1: 1 RBD up, state 2: 2 RBD up, state 3: 1 RBD up and 1 RBD down, and state 4: 2 RBD up and 1 RBD down) (Linsky et al., 2020) which were deposited in the electron microscopy databank and protein data bank, but the structures are not yet available for the public. Eleven residues of the state four trimeric spike protein were determined to interface with CTC-442.2. We compared the 11 residues with the interacting residues identified in our structure and found nine that are interacting residues, Tyr489, Phe486, Gln493, Asn501, Thr500, Tyr449, Phe456, Asn487, and Gln498. The remaining two, Tyr495 and Ala475, do not meet the criterion of 4 Å for interacting resides, but are located in the interface between the trimeric spike protein and ACE2 in our structure, at distances of 4.13 Å and 4.28 Å to ACE2 residues, respectively. Therefore, all the interface residues were confirmed in our structure.

## Conclusions

The structure of the trimeric form of the full-length wild-type spike protein complexed with ACE2 was generated using homology modeling. The homology model was of good quality, as determined by Ramachandran plot evaluation. Using molecular dynamics simulations of the homology model, the residues in the spike protein that are key for tightly binding ACE2 were identified. Most of the interacting residues reported in the crystal structure of the monomeric truncated spike protein RBD bound with ACE2 were among the interacting residues identified in this study with interaction distances defined as ≤4 Å, and all were included when considering those just slighter >4 Å. In addition, one previously excluded residue and five new interacting residues were identified as likely to be important contributors to ACE2 binding. Of the 16 interacting residues analyzed by alanine screening, 15 were confirmed to be important for the binding between the spike protein and ACE2. The binding interactions between the spike protein and ACE2 that were identified using the trimeric form of full-length wild-type spike protein are different from those reported in the crystal structure of the monomeric form of the spike protein RBD, indicating that the binding interface between the spike protein and ACE2 receptor is likely to be dependent on the tertiary structure of the spike protein used in the analysis. Together, the constructed structure of the trimeric full-length wild-type spike protein bound with ACE2 and the key binding residues identified in this study provide new insights into understanding mechanisms of SARS-CoV-2 infection of host cells, which could facilitate the development of drugs and vaccines to prevent SARS-CoV-2 infection and to combat COVID-19.

## Data Availability Statement

The raw data supporting the conclusions of this article will be made available by the authors, without undue reservation.

## Author Contributions

SS, HH, MA, JH, and TP: conceived the experiment. SS and WG: conducted the experiment. SS, BP, GY, and ZJ: analyzed the result. SS, HH, MA, JH, and TP: wrote the manuscript. All authors contributed to the article and approved the submitted version.

## Conflict of Interest

The authors declare that the research was conducted in the absence of any commercial or financial relationships that could be construed as a potential conflict of interest.
