# Supplementary material for: Elucidating Interactions Between SARS-CoV-2 Trimeric Spike Protein and ACE2 Using Homology Modeling and Molecular Dynamics Simulations
Source: Front Chem. 2021 Jan 5;8:622632. doi: 10.3389/fchem.2020.622632 (PMC7813797; doi:10.3389/fchem.2020.622632)
Supplement: Supplementary file 1 [file Table_1.DOCX]

***Supplementary Material***

1 SUPPLEMENTARY FIGURE


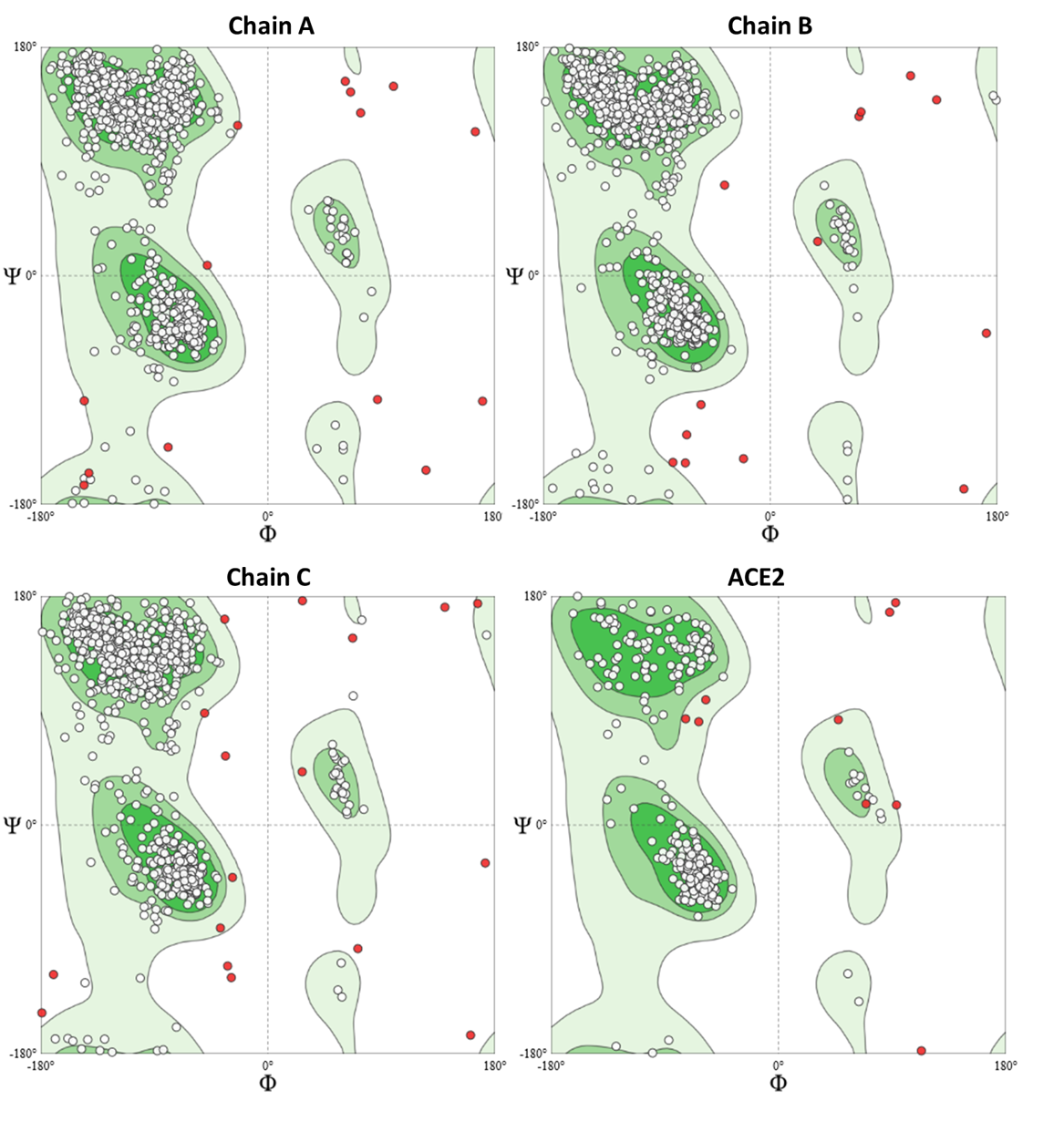


Figure S1. Ramachandran plots of individual chains in the trimeric form of the Severe Acute Respiratory Syndrome Coronavirus-2 spike protein (Chain A, B, and C) and ACE2. The X-axis and Y-axis represent phi and psi in degree, respectively. The favored regions are in green, the allowed regions are in light green, and the outlier regions are in white.
